# Semi‐Quantitative Detection of Respiratory Pathogens: A Systematic Review and Meta‐Analysis of Results From the BIOFIRE FILMARRAY Pneumonia Panel and Culture

**DOI:** 10.1002/mbo3.70086

**Published:** 2025-12-29

**Authors:** Benjamin Hommel, Ophélie Hurtado, Brooklyn Noble, Jay Jones, Florence Allantaz, Tristan T. Timbrook, Gennaro De Pascale, Brunella Posteraro, Maurizio Sanguinetti

**Affiliations:** ^1^ bioMérieux, Marcy l'Étoile France; ^2^ bioMérieux Salt Lake City Utah USA; ^3^ Barnes‐Jewish Hospital St. Louis Missouri USA; ^4^ Dipartimento di Scienze dell'Emergenza Anestesiologiche e della Rianimazione, Fondazione Policlinico Universitario A Gemelli IRCCS Rome Italy; ^5^ Dipartimento di Scienze Biotecnologiche di Base, Cliniche Intensivologiche e Perioperatorie Università Cattolica del Sacro Cuore Rome Italy; ^6^ Dipartimento di Scienze di Laboratorio ed Ematologiche Fondazione Policlinico Universitario A. Gemelli IRCCS Rome Italy

**Keywords:** multiplex PCR, pneumonia, quantitative culture, semi‐quantification

## Abstract

This systematic review and meta‐analysis compared bacterial semi‐quantification of respiratory samples from the BIOFIRE FILMARRAY Pneumonia (PN) Panels with quantitative and semi‐quantitative culture methods (qCMs). Fourteen studies comprising 1,654 samples were included. Across both bronchoalveolar lavage‐like and endotracheal aspirate‐like specimens, the BIOFIRE PN Panel reported consistently higher bacterial loads than qCMs, with pooled mean differences of 1.17 and 0.95 log, respectively. Discrepancies decreased as culture‐reported bacterial burden increased. The concordance rate in identifying the predominant pathogen was 94%, supporting the panel's clinical relevance. However, differential reporting at lower bacterial loads suggests that existing culture‐based thresholds may not translate directly to molecular diagnostics. These findings highlight the need for pathogen‐ and method‐specific interpretive thresholds to optimize the diagnostic utility of semi‐quantitative molecular results and inform antimicrobial stewardship decisions.

## Graphical abstract

The BIOFIRE FILMARRAY pneumonia panel reports semi‐quantitative bacterial loads typically higher than culture, with discrepancies concentrated at low loads, but shows 94% concordance for the predominant pathogen. Findings support PCR‐specific thresholds for interpretation.

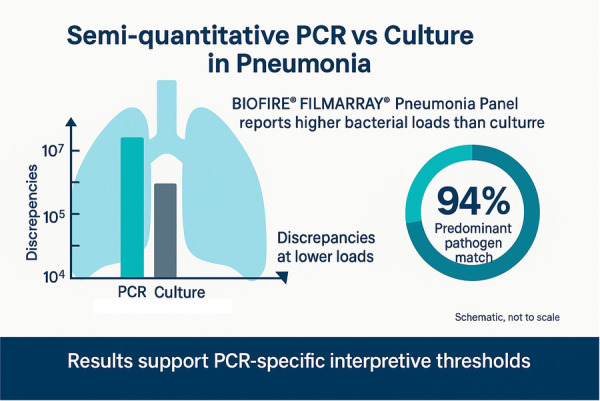

## 1 Introduction

Diagnosing respiratory infections remains a major clinical and microbiological challenge, particularly due to the difficulty in distinguishing colonization from true infection in nonsterile sites. Accurate pathogen identification and quantification in respiratory samples can support clinical decision‐making and improve antimicrobial stewardship.

Quantitative and semi‐quantitative culture methods (qCMs) are recommended by major international guidelines for the diagnosis of hospital‐acquired and ventilator‐associated pneumonia (HAP/VAP), following the original studies that established thresholds discussed by Baselski and Wunderink (Baselski and Wunderink 1994). These recommendations often rely on predefined threshold values to support antibiotic initiation or discontinuation. For instance, the 2016 IDSA/ATS guidelines suggest withholding antibiotics in bronchoalveolar lavage (BAL) samples with < 10⁴ colony‐forming units per milliliter (CFU/mL) or in endotracheal aspirates (ETAs) with < 10⁵ CFU/mL (Kalil et al. 2016). These thresholds aim to increase specificity and reduce unnecessary antibiotic use.

While the IDSA/ATS guidelines favor noninvasive sampling with semi‐quantitative cultures, the ERS/ESICM/ESCMID/ALAT guidelines encourage the use of invasive samples (e.g., BAL or protected specimen brush) and quantitative cultures whenever possible, as they provide higher specificity and are less prone to contamination (Torres et al. 2017). Nevertheless, traditional culture‐based methods are time‐consuming and may be negatively affected by prior antibiotic exposure.

Molecular syndromic panels such as the BIOFIRE FILMARRAY Pneumonia (PN) Panels offer rapid detection of multiple bacterial and viral pathogens, including 15 bacteria reported with semi‐quantitative values in a log scale (10⁴, 10⁵, 10⁶, or ≥ 10⁷ copies/mL). The BIOFIRE PN Panel also detects three atypical bacteria and seven antimicrobial resistance markers. A “*plus*” version of the panel is also available, which includes MERS‐CoV. Its potential to optimize empiric antimicrobial treatment has been documented in several studies (Enne et al. 2025; Virk et al. 2024; Poole et al. 2022) and it is now included in recent guidelines and clinical pathways for severe community‐acquired pneumonia (Martin‐Loeches et al. 2023; Albarillo et al. 2025).

Although this technology provides semi‐quantitative data similar to qCMs, no comprehensive evaluation has yet compared the agreement between molecular and culture‐based semi‐quantification across respiratory sample types.

To address this gap (Walker et al. 2024; Moy et al. 2023), we conducted a systematic review and meta‐analysis comparing bacterial semi‐quantification results obtained with the BIOFIRE PN Panel and BIOFIRE PN Panel *plus* with culture. The aims were: (i) to assess the agreement in semi‐quantification by study and sample invasiveness (BAL, ETA, sputum); (ii) to evaluate differences by pathogen and sample type; and (iii) to explore the correlation in identifying the predominant pathogen between the BIOFIRE PN Panel and qCMs.

## 2 Methods

This review was reported by the Preferred Reporting Items for Systematic Reviews and Meta‐Analyses (PRISMA) guidelines (Page et al. 2021) and registered in https://www.crd.york.ac.uk/prospero/ under: CRD42023468162. This systematic literature review and meta‐analysis was conducted according to Preferred Reporting Items for Systematic Reviews and Meta‐Analyses (PRISMA) guidelines provided in Table S1.

### 2.1 Literature Search Strategy

We searched first EMBASE (from 1946 to July 2023) and Ovid MEDLINE (from 1974 to July 2023) using different search strings into the databases (Mesh terms for Medline and Emtree terms for Embase) such as “respiratory tract infections,” “pneumonia,” “lung,” “pulmonary inflammation,” “multiplex polymerase chain reaction,” “filmarray,” “Biofire,” and “culture.” The query used Boolean operators (“or” “exp” mp.”, etc). A second literature review was performed in parallel through PUBMED and GOOGLE SCHOLAR with a similar approach (from 2018 to 2023) (Figure S1). Two authors (B.H. and O.H.) searched the literature and performed article selection independently. Results were merged, excluding the studies that didn't match the inclusion criteria.

### 2.2 Study Selection and Inclusion Exclusion Criteria

Two reviewers (B.H. and O.H.) decided on the eligibility of a paper to be included according to the inclusion criteria. In case of disagreement, a consensus between the two reviewing analysts permitted its resolution. Duplicates were removed. Inclusion criteria were all full‐text articles that used both detection and semi‐quantification of pathogens with the BIOFIRE PN Panel and quantitative and semi‐quantitative culture methods in respiratory samples with (BAL/BAL‐like or ETA/ETA‐like sampling). The terms ‘ETA‐like’ refer to induced, expectorated sputa, ETA and “BAL‐like” to BAL, mini‐BAL and protected specimen brush (PSB), as detailed in Table S2. It is important to note that, with the exception of PSB, all respiratory sample types are indicated for use by the manufacturer. To be included, articles had to have data on the comparison between culture and BIOFIRE PN Panel methods: semi‐quantification of pathogens with the BIOFIRE PN Panel and quantitative and semi‐quantitative culture methods, information on the sample type used in the study, and pathogen identification. There was no restriction on the patient population (adults or pediatrics), fresh or frozen samples were also included. Studies were excluded if there was no culture quantification or culture without quantification such as rare, few, moderate, heavy and +1, +2, +3, and so forth. Studies with no comparison between molecular and culture semi‐quantification methods were excluded. Systematic reviews, meta‐analyses, case reports, guidelines, conference abstracts/papers or reviews, nonhuman and non‐English studies were not included in the analysis. As the panel launch date was in 2018, papers published before this date were excluded. Finally, authors were contacted for the individual patient data to facilitate additional analyses (e.g., organism specific semi‐quantification differences), and studies were excluded in those for whom authors didn't respond to the data request.

### 2.3 Outcomes

The primary outcome was the semi‐quantification difference between culture and BIOFIRE PN Panel by study and sample type, then the semi‐quantification difference by organism and sample type. Third, the evolution of the difference between the two methods on the measurement scale was evaluated. Finally, the predominance of a pathogen was addressed by comparing the correlation between the predominant bacteria identified by the two methods. For this outcome, a single sample and method can have multiple predominant pathogens if multiple pathogens were detected at the highest semi‐quantification value.

### 2.4 Data Extraction, Curation, and Risk of Bias Assessments

A standardized spreadsheet was sent to the authors of selected publications containing the sample number, sample type, type of pathogen, and quantification with both methods. Data extraction was performed by collecting the first author's name, study year, design, sample number, type, quality, and inclusion method.

The analysis was restricted to bacterial pathogens detected by the BIOFIRE PN Panel. According to the manufacturer, a pathogen is considered detected by the BIOFIRE PN Panel when its semi‐quantification exceeds 10^3.5^ copies/mL. To ensure comparability, an equivalent threshold of 10^3.5^ CFU/mL was applied to define a positive result in culture‐based bacterial detection.

The BIOFIRE PN Panel always reports semi‐quantification in predefined bins, with the highest category being ≥ 10⁷ copies/mL. For consistency, culture quantification values equal to or exceeding this threshold were also categorized as ≥ 10⁷ CFU/mL. Culture semi‐quantitative values were binned using the following criteria:

1. When quantification was reported as a range (*n* = 112), the upper value of the range was used.
2. When values were expressed with inequality symbols (*n* = 545), a midpoint approach was used: values such as ≥ 10⁵ CFU/mL, ≤ 10⁵ CFU/mL, > 10⁵ CFU/mL, or < 10⁵ CFU/mL were all interpreted as 10⁵ CFU/mL.

Two investigators (O.H. and B.H.) completed record screening, eligibility assessment, data extraction, and Quality Assessment of Diagnostic Accuracy Studies (QUADAS) two quality assessments. In case of disagreement, the opinion of an independent third reviewer prevailed. This tool assesses the risk of bias among four domains: index test, patient selection, reference standard, and flow and timing.

### 2.5 Statistical Analysis

The primary outcome was to evaluate the difference between the semi‐quantification bin levels reported by the BIOFIRE PN Panel and the corresponding semi‐quantitative culture values, stratified by sample type and bacterial species. For consistency, culture results were mapped into bin categories aligned with those of the BIOFIRE PN Panel (10⁴, 10⁵, 10⁶, and ≥ 10⁷ CFU/mL). Confidence intervals were calculated using a Student's t‐distribution. Welch's *t*‐test was used to test for significant differences between means. A *p* ≤ 0.05 was considered significant. Data were analyzed using Python 3.11. A random‐effects model was used to estimate pooled semi‐quantification bin differences and 95% confidence intervals for ETA‐like and BAL‐like samples using restricted maximum likelihood estimation. Heterogeneity between studies was evaluated with *I* ^2^ estimation and the Cochran Q test. The metaphor package (version 4.8.0) in R software (version 4.2.2) was utilized for the meta‐analysis.

## 3 Results

**Figure 1:**
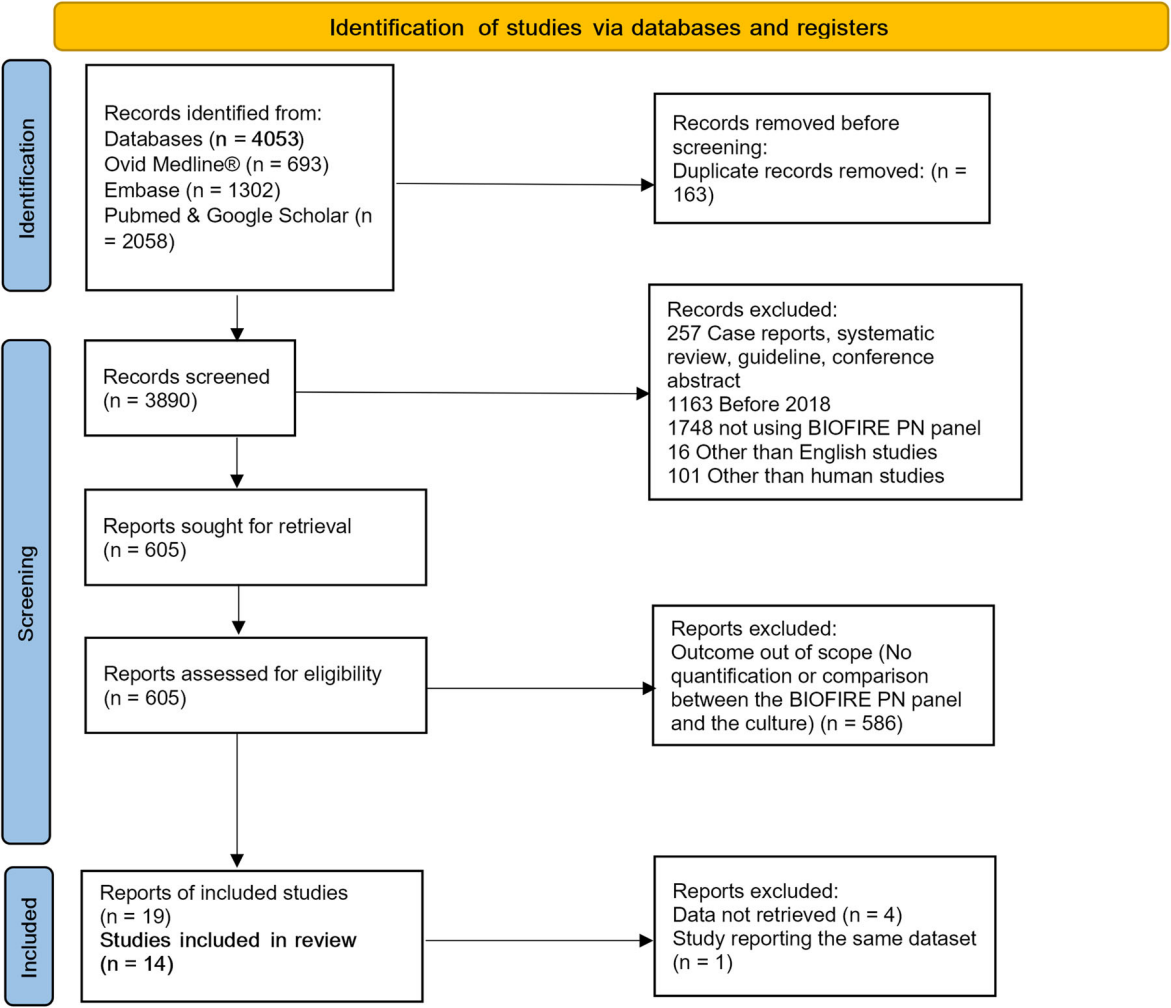
Study selection process and search strategy results from MEDLINE/Embase and PubMed/Google Scholar, following PRISMA guidelines (Page et al. 2021).

Our electronic search identified 4053 articles meeting the initial inclusion criteria; 163 duplicates were removed, 3890 were screened, and 605 were assessed for eligibility. 586 matching the search strings criteria were excluded because no semi‐quantification was assessed by culture, 19 were selected for potential inclusion and due to the availability of data and a doubloon data set, 14 were analyzed (Figure 1) with *n* = 1738 samples included. In terms of quality assessment, most studies had a low risk of bias and a high applicability (Figure S2). Importantly, in most studies, the reference standard was judged to have a higher risk of bias and limited applicability, primarily due to its being performed after the BIOFIRE PN Panel results were available.

The characteristics of the included studies are summarized in Table 1. The studies span multiple countries and employ diverse designs: 10 out of 14 were prospective, and in 10 studies, patients were recruited from intensive care units. A variety of respiratory sample types were analyzed. Notably, 7 out of 14 studies reported antibiotic use before sampling, with some studies reporting pretreatment in up to 91% of patients. All studies focused on suspected lower respiratory tract infections (LRTIs), and 6 of them specifically addressed COVID‐19‐related pneumonia.

**Table 1:** Baseline characteristics of the studies included in the meta‐analysis comparing semi‐quantification results between culture‐based methods and the BIOFIRE PN Panel.

Author, year	Country	Study design	Hospital setting	Sample size included, No.	Patient number included (*n*)	Sample type(s)	Antibiotics before sampling	Pneumonia Classification/Diagnostic
Buchan B.W., 2020	USA	Prospective and retrospective, multicentric	Inpatients	259	259	259 BAL	Yes (% NA)	Suspected LRTI
Camelena F., 2020	France	Prospective, monocentric	ICU	96	43	BAL	Yes (55% of the samples)	Suspected LRTI, COVID‐19
Camelena F., 2021	France	Retrospective, monocentric	ICU	147	92	ETA, BAL, Sputum, PSB	Yes (69% of the samples)	Suspected LRTI, COVID‐19
Cremet L., 2020	France	Prospective, multicentric	ICU	237	100	76 BAL ‐ 161 ETA	Yes (25% of patients)	Suspected LRTI
Edin A., 2020	Sweden	Prospective, monocentric	ICU, Conventional	84	84	16 BAL ‐9 ETA ‐ 59 Sputum	NA	Suspected LRTI
Ferrer J., 2022	Spain	Retrospective, monocentric	ICU	163	109	163 ETA	Yes (91% of patients)	Suspected LRTI COVID‐19
Foschi C., 2021	Italy	Retrospective, monocentric	ICU	230	178	52 BAL ‐ 178 BA	NA	Suspected LRTI COVID‐19
Gadsby N., 2023	UK	Prospective, monocentric	NA	22	22	22 Sputum	No	Hospitalized CAP patients with COPD exacerbation
Ginocchio C., 2021	Austria, Belgium, Denmark, Israel, Italy, France, Germany, Netherlands, Portugal, Spain, Sweden, Switzerland, UK	Prospective multicentric	NA	2476	NA	1234 BAL ‐ 1 242 Sputum/ETA	Yes (% NA)	Suspected LRTI
Mitton B., 2020	South Africa	Prospective, monocentric	ICU, Conventional	59	NA	1 BAL ‐ 58 ETA	NA	Suspected LRTI
Molina FJ., 2022	Colombia	Prospective, multicentric	ICU	110	110	BAL ‐ ETA	NA	Suspected LRTI COVID‐19
Murphy CN., 2020	U.S.A	Prospective, multicentric	In and outpatient, ED	1682	NA	846 BAL ‐ 836 Sputum/ETA	NA	Suspected LRTI
Posteraro B., 2021	Italy	Prospective, monocentric	ICU	212	150	82 BAL ‐ 130 ETA	Yes(%NA)	Suspected LRTI, COVID 19
Stafylaki D., 2022	Greece	Prospective, monocentric	ICU	79 (and 40 pneumonia control patient)	119?	BAL	NA	Suspected LRTI

Abbreviations: BAL, bronchoalveolar lavage; CAP, community‐acquired pneumonia; ETA, endotracheal aspirate; HAP, hospital‐acquired pneumonia; LRTI, low respiratory tract infection; PSB, protected specimen brush (a specimen type not indicated for use in the manufacturer's intended use); VAP, ventilator‐associated pneumonia.

**Figure 2:**
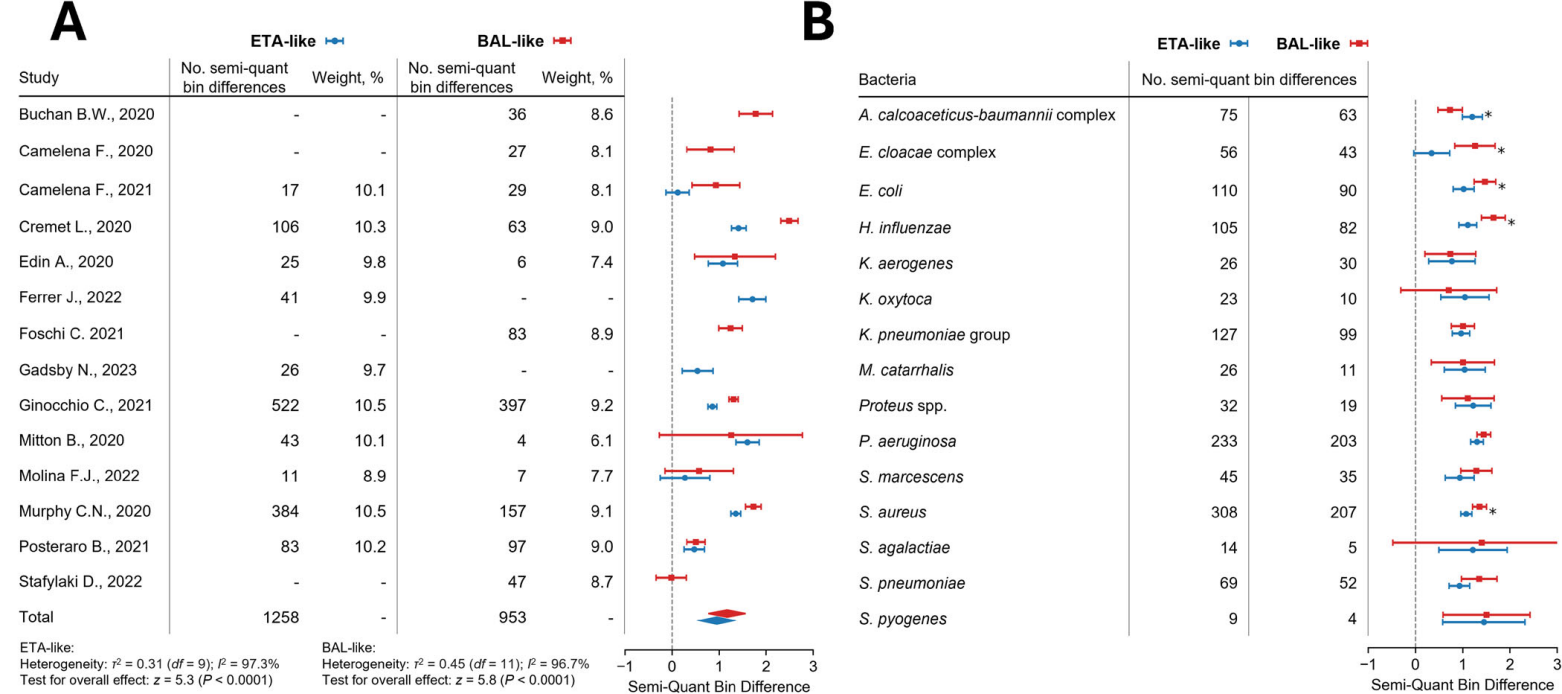
Mean semi‐quantification bin difference per data set and sample type between qCMs and the BIOFIRE PN Panel. BIOFIRE PN Panel reported higher semi‐quantification values compared to culture‐based methods, although with considerable heterogeneity across bacterial species and sample types. (A) A random‐effects model was used and corresponding 95% confidence intervals, based on Restricted Maximum Likelihood (REML) estimation. (B) Mean semi‐quantification bin difference per bacterial species and sample type. Error bars represent 95% confidence intervals calculated using Student's t‐distribution. “Overall” refers to all datasets combined. BAL‐like samples: bronchoalveolar lavage and mini‐bronchoalveolar lavage; ETA‐like samples: induced or expectorated sputum and endotracheal aspirates; qCMs: quantitative/semi‐quantitative culture methods.

The 14 included studies comprised a total of 1654 respiratory samples, each with at least one bacterial species detected semi‐quantitatively by both methods. For analysis, samples were categorized as ETA‐like (including induced and expectorated sputum, and endotracheal aspirates [ETA]) or BAL‐like (including bronchoalveolar lavage [BAL] and mini‐BAL). Regarding the primary outcome, the pooled difference in semi‐quantification, expressed in log₁₀ units, was 0.95 (95% CI, 0.60–1.30) for ETA‐like samples and 1.17 (95% CI, 0.77–1.57) for BAL‐like samples (Figure 2A). These findings indicate that the BIOFIRE PN Panel consistently reported higher semi‐quantification values compared to culture‐based methods, although with considerable heterogeneity across bacterial species and sample types (Figure 2B). A greater semi‐quantification difference in BAL‐like samples compared to ETA‐like samples was observed for *Enterobacter cloacae* complex, *Escherichia coli*, *Haemophilus influenzae*, and *Staphylococcus aureus*. In contrast, *Acinetobacter calcoaceticus–baumannii* complex showed a higher difference in ETA‐like samples. No substantial differences were observed for the remaining bacterial species.

**Figure 3:**
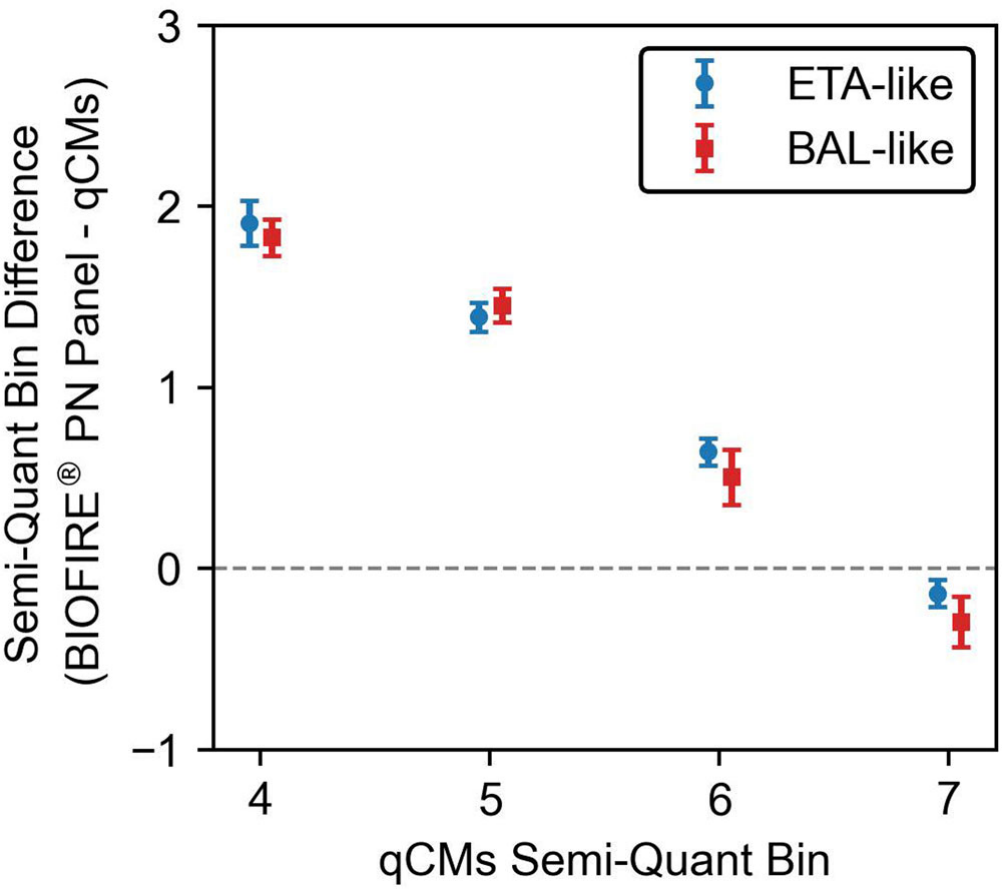
Mean semi‐quantification bin difference (BIOFIRE PN Panel minus qCMs) across qCM measurement bins, stratified by sample type. The BIOFIRE PN Panel consistently reported higher semi‐quantification values compared to culture‐based methods, with the largest differences observed at lower qCM bins (e.g., 10⁴), and a reversal of this trend at higher bins (e.g., 10⁷), regardless of sample type. Error bars represent 95% confidence intervals calculated using Student's t‐distribution. BAL‐like samples: bronchoalveolar lavage and mini‐bronchoalveolar lavage; ETA‐like samples: induced or expectorated sputum and endotracheal aspirates; qCMs: quantitative/semi‐quantitative culture methods.

The trend in the semi‐quantification difference between the two methods across the measurement scale showed that the mean bin difference (BIOFIRE PN Panel minus qCMs) decreased as the qCMs semi‐quantification increased, regardless of sample type. At bin 10⁴, the mean difference, expressed in log₁₀ units, was 1.83 (95% CI, 1.73–1.93) for BAL‐like samples and 1.91 (95% CI, 1.78–2.03) for ETA‐like samples. Conversely, at bin 10⁷, the difference was negative: −0.29 (95% CI, −0.43 to −0.15) for BAL‐like samples and −0.13 (95% CI, −0.21 to −0.06) for ETA‐like samples (Figure 3).

**Figure 4:**
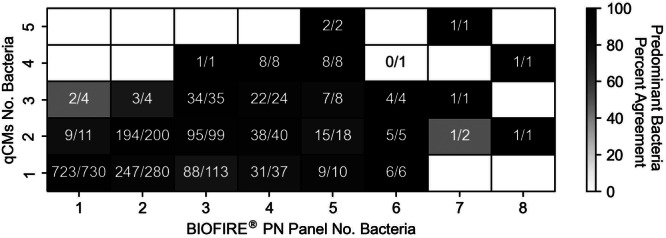
Frequency of concordant predominant bacteria between qCMs and the BIOFIRE PN Panel when multiple bacteria were detected. Values indicate the number of samples in which both methods identified the same predominant bacterial species, based on the number of bacteria detected by the BIOFIRE PN Panel and by qCMs. Percentages are shown in parentheses. The frequency was calculated as: (number of samples with the same predominant bacteria given the number of BIOFIRE PN Panel and qCM bacteria detected)/(total number of samples with that number of BIOFIRE PN Panel and qCM bacteria detected). qCMs: quantitative/semi‐quantitative culture methods.

Overall, the concordance between the BIOFIRE PN Panel and qCMs in identifying the same predominant bacterial species within a given specimen was 94% (1556/1654). This included 99% concordance (723/730) in samples where both methods detected a single bacterial species, and 90% concordance (833/924) in samples with multiple bacteria detected by either or both methods (Figure 4). Among the 98 discrepant samples, the proportion of BAL‐like samples was lower than ETA‐like samples: 29.6% (29/98) versus 70.4% (69/98), respectively. It should be noted that a single specimen and method could report more than one predominant pathogen when multiple species were detected at the highest semi‐quantification value. Among the 108 predominant pathogens identified by the BIOFIRE PN Panel in discrepant cases, the most frequently reported were *H. influenzae* (28/108), *S. aureus* (18/108), and *Moraxella catarrhalis* (16/108). In comparison, qCMs identified 109 predominant pathogens, most commonly *S. aureus* (24/109), *Pseudomonas aeruginosa* (15/109), and *E. coli* (13/109) (Table S3).

## 4 Discussion

This analysis reveals several key findings with practical implications for the diagnosis and management of HAP and VAP in critically ill patients. These include the enhanced sensitivity of molecular diagnostics, an average one‐log difference in semi‐quantification, and a nonlinear agreement between methods—findings that suggest the need to develop specific treatment algorithms and validate thresholds tailored to the BIOFIRE PN Panel. Overall, the results underscore the evolving role of molecular diagnostics and challenge the continued reliance on culture‐based methods as the diagnostic gold standard.

### 4.1 Enhanced Sensitivity of Molecular Diagnostics and Semi‐Quantification Differences

Semi‐quantification data from 14 studies and over 1654 samples indicate that the BIOFIRE PN Panel consistently reports higher bacterial loads than qCM, with mean differences of 0.95 log for ETA‐like and 1.17 log for BAL‐like samples. Importantly, these differences were more pronounced in specific pathogens—*E. coli, H. influenzae*, and *S. aureus* in BAL‐like samples, and *A. baumannii* in ETA‐like samples. The observed differences in semi‐quantification across pathogens and sample types are likely multifactorial. For instance, the higher loads reported for *H. influenzae* and *S. aureus* in BAL‐like samples may reflect a combination of biological factors (e.g., organism growth characteristics) and technical factors (e.g., specimen‐related issues such as reduced contamination in BAL‐like samples) that may drive the observed difference (Hurtado et al. 2024). BAL samples access the distal airways more directly. They are less prone to contamination from upper airway flora compared to ETA, potentially allowing more accurate quantification of lower respiratory tract pathogens. The comparative evaluation of molecular platforms, particularly the BIOFIRE PN Panel, against qCM demonstrates that multiplex PCR assays exhibit superior sensitivity. Enne et al.‘s (Enne et al. 2022) multicenter study, utilizing Bayesian latent class analysis, confirmed that molecular methods achieved performance sensitivities of 83.9%–99.3%, whereas culture‐based methods ranged only between 27.1% and 68.7%. Considering these results, the increased detection of the BIOFIRE PN Panel semi‐quantification may reflect the under‐performance of conventional cultures' semi‐quantification. These findings are aligned with the broader consensus that culture lacks the sensitivity required for timely and accurate pathogen detection, especially following antibiotic initiation. Antibiotic pretreatment, noted in approximately half of the datasets, is a well‐recognized factor that diminishes culture sensitivity by suppressing viable organisms. In contrast, molecular methods remain robust under these conditions (Fratoni et al. 2023), which enhances their diagnostic utility. This resilience supports the early use of PCR diagnostics in patients who have already received empirical therapy, a common scenario in ICU settings.

### 4.2 Pathogen Concordance and Clinical Relevance

A notable finding is the 94% concordance rate between BIOFIRE PN panel and qCM in identifying the predominant bacterial species within a sample. This level of agreement reinforces the clinical utility of molecular diagnostics as an aid in determining the etiologic agent, even in polymicrobial samples where interpretation can be challenging. The semi‐quantification data can thus aid clinicians in distinguishing true pathogens from colonizers, a crucial aspect in guiding targeted therapy.

### 4.3 Nonlinear Agreement and Threshold Considerations

The analysis revealed a nonuniform distribution of semi‐quantification differences across the detection scale. Discrepancies were more pronounced at lower pathogen loads (e.g., bin 10⁴), while agreement between PCR and culture improved at higher loads (e.g., bin 10⁷). This pattern suggests that concordance between methods increases with higher bacterial burden but decreases near the diagnostic threshold—precisely where clinical decisions regarding the initiation or discontinuation of antibiotics are most challenging.

This has implications for current diagnostic thresholds. For instance, guidelines often use the 10⁴ CFU/mL cutoff in BAL to guide antibiotic discontinuation. Retrospective evidence, such as the study by Raman et al (Raman et al. 2013)., compared early versus late discontinuation of antibiotics in patients with culture‐negative quantitative bronchoscopy cultures in VAP (< 10⁴ CFU/mL). The adjusted multivariable analysis showed no significant difference in mortality (25.0% vs. 30.6%) but did demonstrate significantly fewer overall, respiratory, and multidrug‐resistant superinfections in the early discontinuation group.

Applying culture‐based thresholds to molecular results may be problematic, as BIOFIRE PN Panel often detects higher loads for the same clinical samples. Candel et al. in an expert opinion paper (Candel et al. 2024) recommend reporting the quantitative results and state that high bacterial burdens—typically ≥ 10⁶ or 10⁷ copies/mL—are generally indicative of a causative pathogen. Yet, lower levels (10⁴–10⁵ copies/mL) may also suggest clinical relevance, particularly for pathogens such as *Pseudomonas aeruginosa*, *Acinetobacter baumannii*, and methicillin‐resistant *Staphylococcus aureus*, especially in patients already receiving appropriate antimicrobial therapy. Furthermore, the detection of multiple pathogens, especially in qualitative‐only reports, warrants cautious interpretation. While the presence of more than two targets can complicate decision‐making, high genomic burdens (≥ 10⁶), even in polymicrobial results, may still support causality. Therefore, interpreting semi‐quantitative PCR findings requires individualization and clinical contextualization (Candel et al. 2024).

These considerations highlight the need for PCR‐specific diagnostic or de‐escalation thresholds, validated through clinical outcome studies, to safely and effectively guide antimicrobial therapy.

### 4.4 Limitations

An important point is that only the corresponding semi‐quantification was evaluated, without considering that potentially positive findings on the BIOFIRE PN Panel may have been recorded as negative in culture. This represents a missing element in our data set and could impact on the interpretation of discrepancies between methods. This systematic review did not include individual clinical data, such as antibiotic use or treatment decisions. Therefore, we were unable to assess the clinical impact of discordant findings between molecular and culture‐based methods. Future studies should integrate diagnostic results with clinical outcomes to address this gap. Additionally, a major limitation of culture‐based methods lies in the variability of laboratory practices and the heterogeneity of quantification techniques, which can compromise the reproducibility and comparability of results across different studies (Prinzi et al. 2021). Finally, the high proportion of patients with severe disease or severe pneumonia in the data set may limit the generalizability of our findings to less severe cases or to broader adult populations. Further research is necessary to assess the validity of these findings in other clinical contexts.

## 5 Conclusions

The integration of the BIOFIRE PN Panel into routine practice must be accompanied by further research to establish clinically relevant thresholds, enhance interpretability, and guide stewardship strategies. Until such standards are established, clinicians should apply molecular diagnostics as part of a multifaceted diagnostic approach, within a comprehensive clinical framework, incorporating patient risk factors, symptoms, local ecology, previous antibiograms, imaging, and treatment history. While PCR offers compelling advantages in sensitivity and speed, its implementation into clinical practice must be accompanied by standardized protocols and stewardship oversight.

## Author Contributions

**Benjamin Hommel:** conceptualization (lead), writing – original draft (supporting), formal analysis (lead), writing – review and editing (equal). **Ophélie Hurtado:** conceptualization (supporting), writing – original draft (supporting), formal analysis (supporting), writing – review and editing (equal). **Brooklyn Noble:** conceptualization (supporting), writing – original draft (supporting), formal analysis (supporting), writing – review and editing (equal). **Jay Jones:** conceptualization (supporting), writing – original draft (supporting), formal analysis (supporting), writing – review and editing (equal). **Florence Allantaz:** conceptualization (supporting), writing – original draft (supporting), formal analysis (supporting), writing – review and editing (equal). **Tristan T. Timbrook:** conceptualization (supporting), writing – original draft (supporting), formal analysis (supporting), writing – review and editing (equal). **Gennaro De Pascale:** conceptualization (supporting), writing – original draft (supporting), formal analysis (supporting), writing – review and editing (equal). **Brunella Posteraro:** conceptualization (supporting), writing – original draft (supporting), formal analysis (supporting), writing – review and editing (equal). **Maurizio Sanguinetti:** conceptualization (supporting), writing – review and editing (equal).

## Ethics Statement

The authors have nothing to report.

## Conflicts of Interest

The authors declare no conflicts of interest.

## Supporting information


**Supporting Figure 1:** Search strategies used for systematic literature review. Detailed search strategies were implemented across multiple databases to identify relevant studies in Ovid MEDLINE and Embase. Additional searches were performed in Google Scholar and PubMed to capture potentially missed studies.

**Supporting Figure 2:** QUADAS‐2 assessment of the risk of bias in studies included in the meta‐analysis comparing semi‐quantification between conventional culture and the BIOFIRE® FILMARRAY® Pneumonia Panel. This figure summarizes the proportion of studies categorized by risk of bias across four domains: patient selection, index test, reference standard, and flow and timing. The top right panel reflects the assessment based on signaling questions within each domain. The bottom right panel presents concerns regarding applicability, based on the extent to which each study addresses the predefined meta‐analysis question.

**Supporting Table 1:** PRISMA 2020 Checklist: This table outlines the PRISMA 2020 guidelines for systematic reviews, detailing each checklist item and its corresponding location in the document.

**Supporting Table 2:** Classification of sample types by respiratory specimen category.

**Supporting Table 3:** Comparative analysis of predominant pathogens identified by BIOFIRE® PN Panel and qCMs. qCMs: quantitative/semi‐quantitative culture methods.
