# Supplementary figures and images for: Semi‐Quantitative Detection of Respiratory Pathogens: A Systematic Review and Meta‐Analysis of Results From the BIOFIRE FILMARRAY Pneumonia Panel and Culture
Source: Microbiologyopen. 2025 Dec 29;15(1):e70086. doi: 10.1002/mbo3.70086 (PMC12748513; doi:10.1002/mbo3.70086)

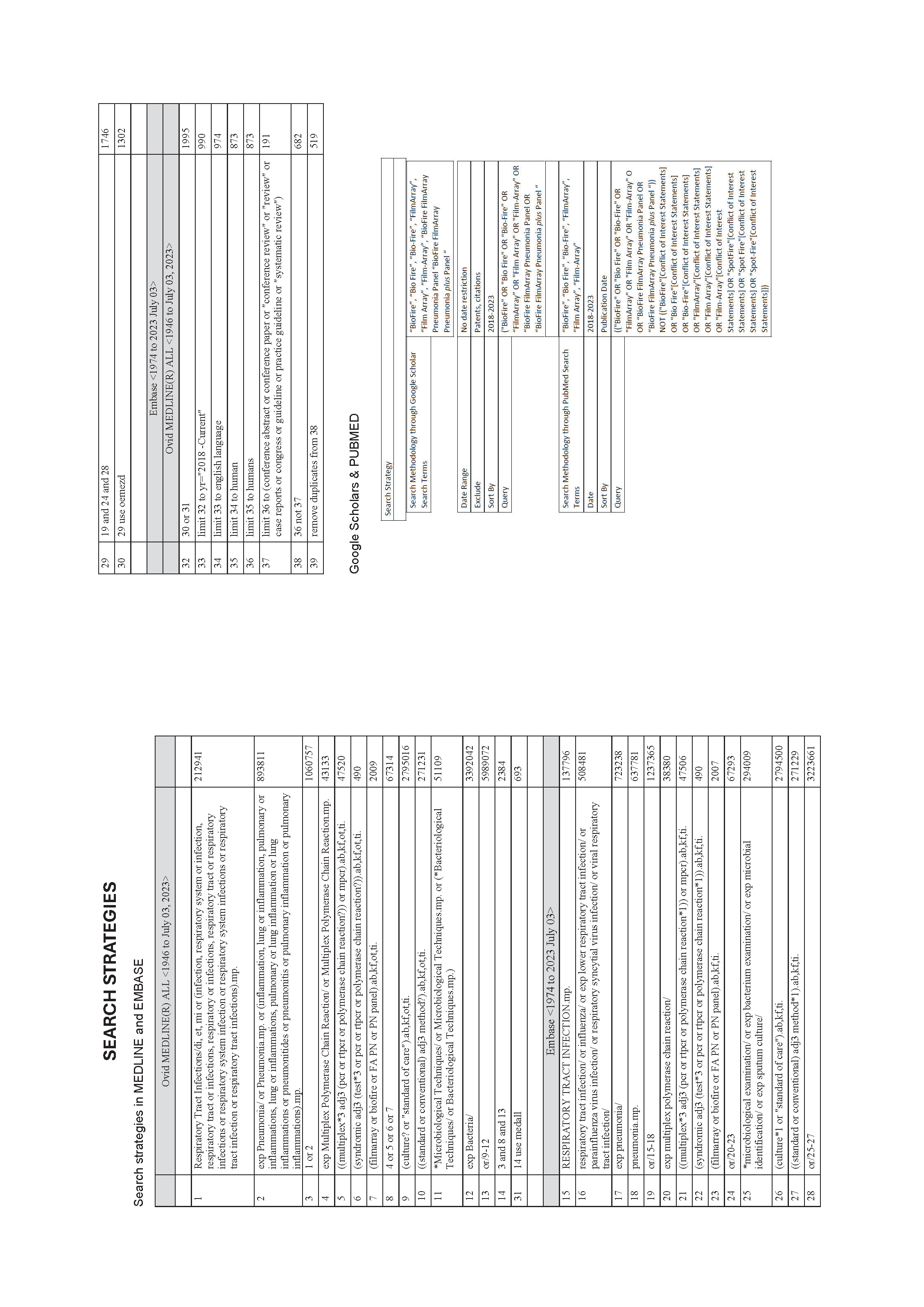

Supplement: Supplementary file 1 — Supporting Figure 1: Search strategies used for systematic literature review. Detailed search strategies were implemented across multiple databases to identify relevant studies in Ovid MEDLINE and Embase. Additional searches were performed in Google Scholar and PubMed to capture potentially missed studies. [file MBO3-15-e70086-s001.tiff]

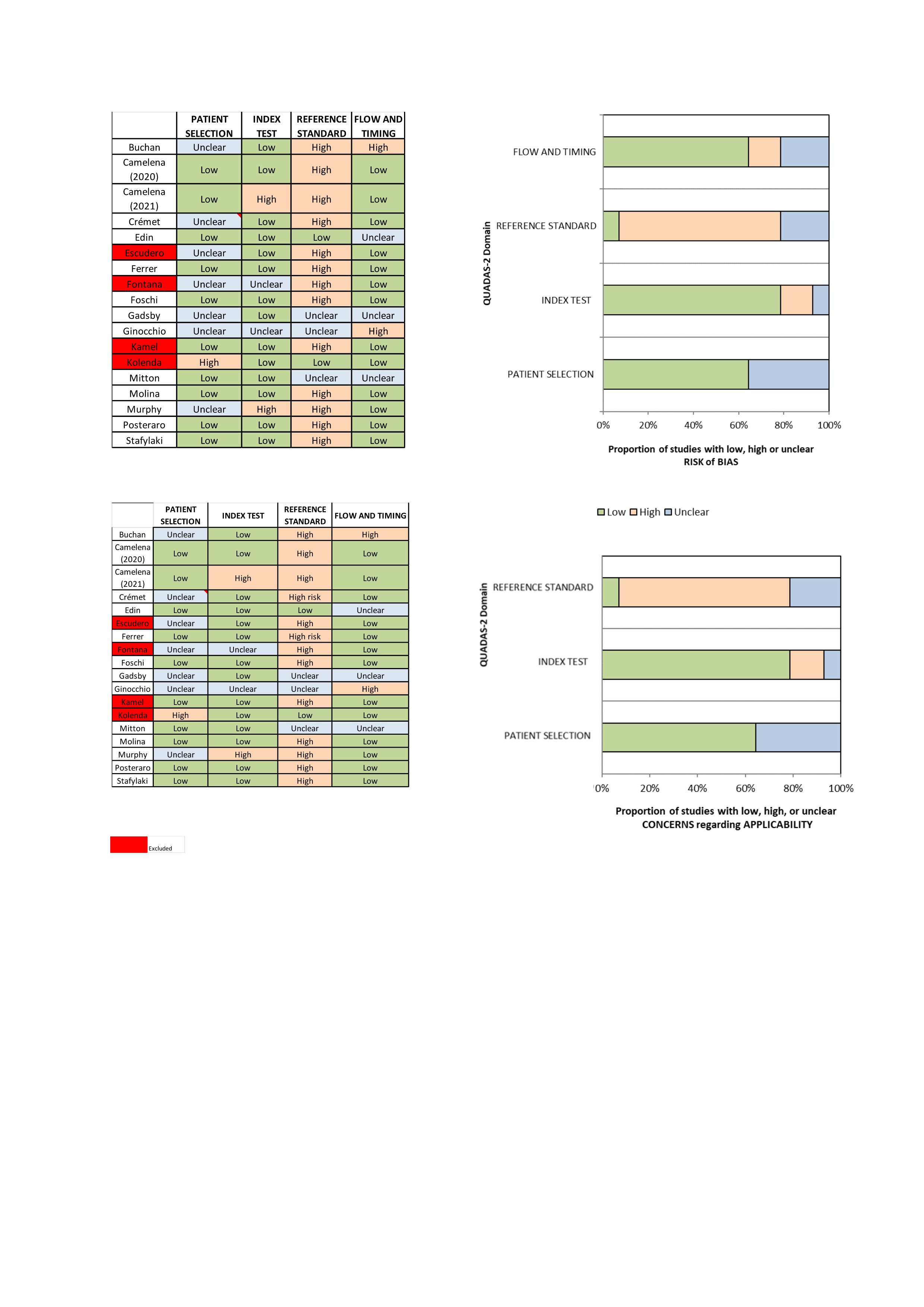

Supplement: Supplementary file 2 — Supporting Figure 2: QUADAS‐2 assessment of the risk of bias in studies included in the meta‐analysis comparing semi‐quantification between conventional culture and the BIOFIRE® FILMARRAY® Pneumonia Panel. This figure summarizes the proportion of studies categorized by risk of bias across four domains: patient selection, index test, reference standard, and flow and timing. The top right panel reflects the assessment based on signaling questions within each domain. The bottom right panel presents concerns regarding applicability, based on the extent to which each study addresses the predefined meta‐analysis question. [file MBO3-15-e70086-s002.tiff]
