# Supplementary material for: Semi‐Quantitative Detection of Respiratory Pathogens: A Systematic Review and Meta‐Analysis of Results From the BIOFIRE FILMARRAY Pneumonia Panel and Culture
Source: Microbiologyopen. 2025 Dec 29;15(1):e70086. doi: 10.1002/mbo3.70086 (PMC12748513; doi:10.1002/mbo3.70086)
Supplement: Supplementary file 4 — Supporting Table 2: Classification of sample types by respiratory specimen category. [file MBO3-15-e70086-s003.docx]

Supplementary Table 2

| Category | Included Sample Types |
| --- | --- |
| ETA-like | Induced sputum, Expectorated sputum,  Endotracheal aspirate (ETA) |
| BAL-like | Bronchoalveolar lavage  (BAL), Mini-BAL, Protected specimen brush (PSB) |
