# Supplementary material for: Semi‐Quantitative Detection of Respiratory Pathogens: A Systematic Review and Meta‐Analysis of Results From the BIOFIRE FILMARRAY Pneumonia Panel and Culture
Source: Microbiologyopen. 2025 Dec 29;15(1):e70086. doi: 10.1002/mbo3.70086 (PMC12748513; doi:10.1002/mbo3.70086)
Supplement: Supplementary file 5 — Supporting Table 3: Comparative analysis of predominant pathogens identified by BIOFIRE® PN Panel and qCMs. qCMs: quantitative/semi‐quantitative culture methods. [file MBO3-15-e70086-s004.docx]

Supplementary Table 3

|  | Predominant pathogens identified by BIOFIRE PN panel | Predominant pathogens identified by qCMs |
| --- | --- | --- |
| *Streptococcus pneumoniae* | 12 | 8 |
| *Staphylococcus aureus* | 18 | 24 |
| *Streptococcus agalactiae* | 5 | 3 |
| *Streptococcus pyogenes* | 2 | 0 |
| *Haemophilus influenzae* | 28 | 6 |
| *Klebsiella oxytoca* | 1 | 3 |
| *Klebsiella pneumoniae group* | 1 | 9 |
| *Escherichia coli* | 3 | 13 |
| *Moraxella catarrhalis* | 16 | 1 |
| *Acinetobacter calcoaceticus-baumannii complex* | 4 | 2 |
| *Serratia marcescens* | 3 | 9 |
| *Enterobacter cloacae complex* | 4 | 9 |
| *Klebsiella aerogenes* | 0 | 3 |
| *Proteus spp.* | 1 | 4 |
| *Pseudomonas aeruginosa* | 10 | 15 |
| Total | **108** | **109** |
